# Supplementary material for: Imaging Proteins Sensitive to Direct Fusions Using Transient Peptide–Peptide Interactions
Source: Nano Lett. 2023 Nov 2;23(22):10633–41. doi: 10.1021/acs.nanolett.3c03780 (PMC10683072; doi:10.1021/acs.nanolett.3c03780)
Supplement: Supplementary file 2 — nl3c03780_si_002.pdf [file nl3c03780_si_002.pdf]

Supporting Information

Imaging proteins sensitive to direct fusions using transient peptide-peptide interactions

Zoe Gidden[1,2, ‡], Curran Oi[3, ‡], Emily J. Johnston[1,4], Zuzanna Konieczna[2,5], Haresh Bhaskar[1,2,5], Lorena Mendive-Tapia[5,6], Fabio de Moliner[5,6], Susan J. Rosser[1,4], Simon G. J. Mochrie[7,8], Marc Vendrell[5,6], Mathew H. Horrocks[2,5,], Lynne Regan[1,4,8,*]*

[1]School of Biological Sciences, University of Edinburgh, Edinburgh, EH9 3DW, United Kingdom, [2]EaStCHEM School of Chemistry, The University of Edinburgh, Edinburgh, EH9 3FJ, United Kingdom, [3]Department of Genome Sciences, University of Washington, Seattle, WA 98195, United States, [4]Centre for Engineering Biology, University of Edinburgh, Edinburgh EH9 3BD, United Kingdom, [5]IRR Chemistry Hub, Institute for Regeneration and Repair, The University of Edinburgh, Edinburgh, EH16 4UU, United Kingdom, [6]Centre for Inflammation Research, The University of Edinburgh, Edinburgh, [7]Department of Physics, Yale University, New Haven, CT 06520, United States, [8]Integrated Graduate Program in Physical and Engineering Biology, Yale University, New Haven, CT 06520, United States, [9]Institute of Quantitative Biology, Biochemistry and Biotechnology, Edinburgh, EH9 3FF, United Kingdom

‡ Z.G. and C.O. contributed equally to this work

*Correspondence and requests for materials should be addressed to L.R. (email: lynne.regan@ed.ac.uk) M.H.H (email: mathew.horrocks@ed.ac.uk)

Materials and Methods	
Figure S1	Representative structures of interacting peptide pairs used in this study.
Figure S2	Can1 function is not impaired by the 101B tag used for LIVE-PAINT.
Figure S3	Gap1 and Bap2 function is not impaired by the 101B tag used for LIVE-PAINT.
Figure S4	101A-mNeonGreen alone does not localize to the plasma membrane.
Figure S5	Full-cell super-resolution images of membrane proteins labelled with the 101A/B peptide pair shown in Figure 3.
Figure S6	Analysis of whole population protein cluster data for each membrane protein imaged in Figure 3.
Figure S7	Quantification of the number of localizations per cluster for each membrane protein imaged in Figure 3.
Figure S8	Histograms of photon counts for SR images of each membrane protein presented in Figure 3.
Figure S9	Localizations are detected throughout three minutes of imaging with LIVE-PAINT.
Figure S10	Representative plots showing localizations classified as membrane, cellular or external for membrane proteins labelled with the 101A/B peptide pair.
Figure S11	KQTSV can be used as a peptide label for LIVE-PAINT imaging with two tandem repeats of PDZ3 fused to mNeonGreen.
Figure S12	Graphs of diffusion lag time as a function of PDZ concentration for the 1xPDZ and 2xPDZ proteins binding to the KQTSV peptide.
Figure S13	Full-cell super-resolution images of membrane proteins labelled with the KQTSV/2xPDZ3 peptide-protein pair shown in Figure 4.
Figure S14	Representative plots showing localizations classified as membrane, cellular or external for membrane proteins labelled with the KQTSV/2xPDZ3 peptide-protein pair.
Table S1	Membrane transporter proteins tagged and imaged in this study.

Table S2	Summary statistics of super-resolution images of membrane associated proteins imaged using LIVE-PAINT in Figure 3.
Table S3	Summary statistics of super-resolution images of membrane associated proteins imaged using LIVE-PAINT in Figure 4.
Table S4	Summary statistics of super-resolution images of Arc35 and Pil1 imaged using two color LIVE-PAINT and shown in Figure 5.
Table S5	Constructs used to measure binding kinetics *ex vivo*.
Table S6	Primers for tagging membrane proteins at their genomic loci.
Table S7	Primers for generating a yeast strain with two membrane-associated proteins tagged at their genomic loci.
Table S8	List of yeast strains used in this study.
References	

MATERIALS AND METHODS

Molecular biology

All cloning was performed in TOP10 *E. coli*, using standard techniques. All constructs were generated in the pFA6a-His3MX6 and pFA6a-KanMX6 yeast integration vectors. All linker sequences used are GGSGSGLQ. Plasmids were constructed via Gibson assembly using NEBuilder® HiFi DNA Assembly Master Mix (New England Biolabs) by amplifying the plasmid backbone via PCR and inserting other parts of the construct either using gBlocks (Integrated DNA Technologies) or other PCR amplified sequences.

Yeast strain construction

Except where otherwise noted, standard methods for genetically modifying yeast and preparing growth media were used.[1]

To label membrane transporter proteins we used the 101A/101B coiled coil interaction pair,[2] which we have previously demonstrated to be compatible with LIVE-PAINT imaging.[3] At the gene level, 101B was fused to the C-terminus of the target membrane protein at its endogenous locus. 101A was fused to mNG, under control of the galactose-inducible promoter, and integrated into the genome, replacing *GAL2*. When *GAL2* is deleted, the galactose-inducible promoter has a linear response with increasing galactose concentration.[4] This same approach was used when tagging Pma1 using the KQTSV/PDZ3 interaction pair.

Yeast strains were produced by amplifying the insert and selection marker from a yeast integration plasmid (pFA6a-His3MX6 or pFA6a-KanMX6) with ~45 bp overhang sequences matching the 45 bp upstream of the target protein's stop codon and ~45 bp downstream of the desired integration site in the genome. The primers used to tag the various proteins studied in this work are listed in Supplementary Table 1. To transform the yeast, a 3 mL culture of yeast was grown overnight in YPD, back-diluted to an OD_{600} of 0.1 in 3 mL in the morning and grown to OD_{600} of ~0.6-0.8. Cells were pelleted, washed with 300 µL of 0.1 M LiAc twice, then pelleted and resuspended in 30 µL of 0.1 M LiAc. Then, with the cells on ice, the following were then added to the cells in order: 100 µL 50% w/v PEG 3350, 15 µL 1.0 M LiAc, 6 µL 7 mg/mL ss carrier DNA, 18 µL DMSO, 15 µL PCR product, and 14 µL sterile water. This was mixed and incubated in a GeneAMP™ PCR System 9700 (Thermofisher Scientific) for 30 minutes at 30˚C, followed by 15 minutes at 42˚C. Cells were pelleted, the transformation buffer was then removed by aspiration, and cells were resuspended in 100 µL water pre-warmed to 30˚C. All 100 µL of cells were then spread on an agar plate containing the appropriate selection. For histidine selection, plates were made from synthetic complete media lacking histidine. For G418 selection, cells were first plated on a YPD plate and then replica plated to a YPD plate including 600 µg/mL G418 the

next morning. Plates were incubated at 30˚C for 2-3 days. A full list of strains used in this study are listed in Supplementary Table 2. Insertion of the desired construct in the genome was checked via colony PCR.

Preparing cells for microscopy

To prepare cells for microscopy, a single colony was picked from an agar plate into 500 µL synthetic complete media. 100 µL of this cell suspension was then pipetted into 400 uL synthetic complete media, to obtain a 1:5 dilution as well. The cells were grown for 16-20 hrs in a shaking incubator at 30 ˚C. Whichever culture had an approximate OD_{600} of 0.1-0.8 was then used for imaging.

To prepare slides for imaging, 22x40 mm glass coverslips with thickness no. 1 (VWR) were cleaned using a 40-minute exposure to argon plasma in a 2.6 L Zepto plasma laboratory unit (Diener Electronic). Frame-Seal slide chambers (9 × 9 mm2, Biorad, Hercules, CA) were attached to the plasma-cleaned coverslips and the surface treated with 100 µL concanavalin A (Sigma-Aldrich) (2 mg/mL in PBS). The concanavalin A was removed from the surface after 30 s by aspirating with a pipette. 100 µL of prepared yeast culture was then pipetted onto the slide and left for approximately 5 minutes to allow cells to attach. The cells were then aspirated from the slide and the surface washed using 100 µL fresh PBS and gently pipetting up and down. 100 µL fresh PBS was then added to the slide before imaging.

TIRF microscopy

Microscopy data was primarily collected using a commercial TIRF microscope (Oxford Nanoimager). Images were acquired with an exposure of 50 ms for between 2000 and 4000 frames using the NimOS software. mNeonGreen was activated using the 488 nm laser at 15% power and

the angle of illumination (TIR angle) was set at 48.5° for all acquisitions. Pixel length was 117 nm. All images were collected at room temperature.

Two color, Cdc12 and Pil1 microscopy data was collected using on a custom built TIRF microscope. mNG was excited using a 488 nm laser (Cobolt MLD 488-200 Diode Laser System, Cobalt, Sweden) ~40W/cm^2 and mCherry was excited using a 561 nm laser (Cobolt DPL Series 561-100 DPSS Laser System, Cobolt, Sweden) ~25W/cm^2. Both lasers were aligned and directed parallel to the optical axis at the edge of a 1.49 NA TIRF objective (CFI Apochromat TIRF 60XC Oil, Nikon, Japan), mounted on an inverted Nikon TI2 microscope (Nikon, Japan). To prevent z-stage drift during imaging a perfect focus system was utilized. Fluorescence collected by the same objective was separated from the returning TIR beam by a dichroic mirror (Di01-R405/488/561/635 (Semrock, Rochester, NY, USA)), and was passed through appropriate filters (488 nm: BLP01-488R, FF01-520/44 (Semrock, NY, USA), 561 nm: LP02-568- RS, FF01-587/35 (Semrock, NY, USA)). Fluorescence was then passed through a 2.5× beam expander and recorded on an EMCCD camera (Delta Evolve 512, Photometrics) operating in frame transfer mode (EMGain = 11.5 e- /ADU and 250 ADU/photon). Each pixel was 103 nm in length. The microscope was automated by using the open-source microscopy platform Micromanager. For two color samples, images were recorded by collecting 200 frames of 50 ms using the 561 nm laser followed by 200 frames of 50 ms using the 488 nm laser. This was repeated 10 times to result in a total of 2,000 frames for both mCherry and mNeonGreen. All Cdc12 and Pil1 images, were acquired using the 488 nm laser using an exposure of 50 ms for between 2000 and 4000 frames. Prior to two color imaging, tetraspeck beads immobilized on a glass coverslip were used to check the image registration and alignment between the two channels. All images were collected at room temperature.

Confocal microscopy

For localization of Pma1 throughout entire cells, a Zeiss LSM880 confocal microscope (with alpha Plan-Apochromat 100x/1.46 Oil DIC M27 Elyra objective) and Airyscan was used to capture mNG fluorescence data (ex/em 488/522) with z-stack increments of 0.170 micron. Airyscan image processing for generation of super-resolution 3D data was carried out in Zen Blue (Zeiss).

Super-resolution analysis

Super-resolution images were analyzed using Fiji (Java 8 2017 release). Single localizations were processed using the Peak Fit function of the Fiji GDSC SMLM plugin, using a signal strength threshold of 30, a minimum photon threshold of 100, and a precision threshold of 15-30 nm. All localization files are available at: https://doi.org/10.5281/zenodo.8101098. The overall resolution of the images was calculated using Fourier Ring Correlation analysis using the Python code available at: https://doi.org/10.5281/zenodo.7275952.

Quantification of membrane specific localizations

First, cell segmentation on the maximum intensity projections was performed using an adaptive Otsu algorithm on CellProfiler.[5] Segmented cell objects were shrunk down by 4 pixels and subtracted from the cell mask to generate membrane masks. The code is available at https://doi.org/10.5281/zenodo.7817411 was then used to tag localizations contained within localization files generated through super-resolution analysis described previously as cell, membrane or external based on overlap with the respective masks. Cells with fewer than 50 localizations were excluded from further analysis.

Cluster analysis

DBSCAN[6] complied in Python 3.8 using epsilon = 0.8 pixels and a minimum points threshold of 3 was used to identify clusters of localizations in the localization files generated through super-resolution analysis described previously. For cells with more than 10 clusters, several parameters were then calculated and recorded: the number of localizations within each cluster, the ID of the nearest cluster, and the distance to the nearest cluster (determined using unsupervised nearest neighbors learning with the Ball Tree algorithm). The eccentricity of the cluster and the maximum length of the cluster was also calculated by fitting a 2 SD confidence ellipse to the cluster. The eccentricity was determined using the following formula:

$$e = \frac{\sqrt{a^2 + b^2}}{a}$$

where a is the semi-major axis length and b is the semi-minor axis length. The maximum length of the clusters was taken to be the major axis length. This information was collated for all images of cells with the same protein target and peptide pair used for labelling. The Python code is available at: https://doi.org/10.5281/zenodo.8359460.

Two color cluster analysis

For two color images, cluster analysis was carried out on localization data for each color as described previously. The distance between each mCherry (Pil1) cluster and the nearest mNeonGreen (Arc35) cluster in the same cell was determined by calculating the Euclidian distance to all the mNG clusters in the cell and recording the shortest distance. This information was collated for all two-color images of Arc35 and Pil1. The Python code is available at: https://doi.org/10.5281/zenodo.8060654.

Localization rate analysis

Localization files generated through super-resolution analysis, described previously were loaded into Python 3.8. The localizations were then plotted to enable identification of localizations from neighboring cells which were then excluded from further analysis. The localizations recorded over intervals of 600 frames (30 s) were totaled for each localization file. The Python code is available at: https://doi.org/10.5281/zenodo.8360920. The total localizations recorded in each 30 s interval were then plotted against total imaging time using GraphPad (Prism).

Determining binding kinetics of peptides *ex vivo*

In order to quantify the binding of PDZ peptide to its target, Fluorescence Correlation Spectroscopy (FCS) was used together with an ex vivo system: a chemically synthesized and fluorescent-labelled KQTSV peptide and PDZ domain proteins (two constructs: 1xPDZ domain-biotin, 2xPDZ domain-biotin, purified from *E. Coli*, Table S5).

SeNBD was incorporated as a small fluorescent amino acid, previously described, and used by de Moliner *et al.*, to ensure unperturbed binding of the KQTSV sequence to the PDZ domain.[7] The peptide was incubated with a range of protein concentrations (for 1xPDZ: 0, 0.5, 4, 8, 12, 16 µM, and for 2xPDZ: 0, 0.5, 4, 8, 12, 16, ~20 µM). All solutions were prepared in triplicates. The final peptide concentration was kept constant at 100 nM.

The diffusion through a confocal volume was observed using a home-built single molecule confocal instrument, previously described by Chappard *et al.*.[8] The confocal volume was illuminated by a 488 nm laser (LBX-405-100-CSB-OE, Oxxius; 1.2 mW irradiation at the back-port), and 5x 30 second traces were recorded for each sample. The bursts of fluorescence as the peptide/peptide-protein passed the confocal volume were recorded on Avalanche Photodiode (APD) detector (PerkinElmer).

A custom-written Python script was used to transform the burst data into autocorrelation curves, and further fit diffusion lag time and diffusion coefficient (https://doi.org/10.5281/zenodo.8388699). Further analysis was performed in GraphPad (Prism). The red channel diffusion lag time data was averaged for each condition (N=3, or N=2 for 1xPDZ where the residual value for a repeat exceeded the other residuals by an order of magnitude), and the standard deviation between repeat measurements calculated. The resulting plots were fit using a non-linear regression function (binding kinetics, one site specific binding) to the following function:

$$y = \frac{B_{max} \cdot x}{K_D + x}$$

where B_{max} is the max diffusion lag time (plateau) and K_D is the equilibrium dissociation constant.

Three fits were performed to obtain the average K_D, using the mean from the three measurements and the values obtained on either extreme (+/- standard deviation).

Growth rate experiments

For growth studies, 100 μL microplate cultures were incubated in Tecan Sunrise plate readers at 30 °C, with 'normal' shaking level, and A_{595} was recorded at 15 min intervals, which correlates with cell density. Cells from pre-cultures (5 mL 6.7 g/L Yeast Nitrogen Base Sigma Y0626, 790 mg/L Complete Supplement Mixture Formedium DCS001, 2 % glucose) were washed with sterile water, and inoculated into test media at starting OD_{600} 0.2 (corresponding to A_{595} 0.04 in the plate reader). For canavanine (SLS C9758) toxicity studies, Complete Supplement Mixture –ARG (Formedium DCS0051) was used. The average OD_{600} measured in blank wells was subtracted from the OD_{600} recorded for the cell cultures and the blank corrected OD_{600} was plotted against incubation time using GraphPad (Prism).

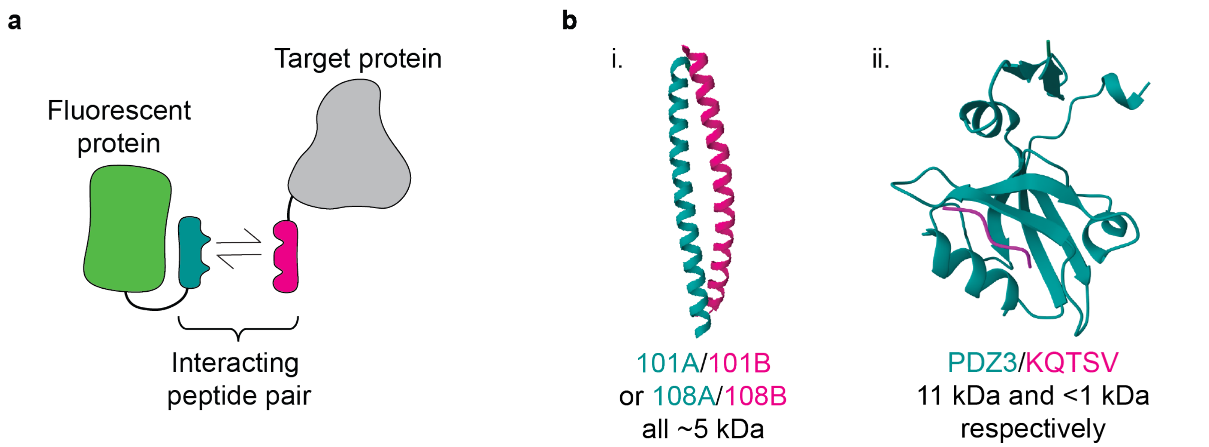

Figure S1. Representative structures of interacting peptide pairs used in this study. (a) Schematic representation of the LIVE-PAINT labelling strategy used in this paper. The fluorescent protein is labelled with a protein or peptide (teal) via a short GS linker. The target protein is labelled with a peptide (pink) also via a short GS linker. (b) Representative crystal structures of the peptide interaction pairs used in this study. (bi) Ribbon structure diagrams for the antiparallel coiled coil peptide pairs, 101A (teal)/101B (pink) and 108A (teal)/108B (pink). The diagram was generated using the PDB file for a similar antiparallel coiled-coil pair SYNZIP2/SYNZIP1 (PDB ID: 3HE5). (bii) Ribbon structure diagram for the PDZ3 (teal)/KQTSV (pink) pair. The diagram was generated using the PDB file for this pair (PDB ID: 1BE9).

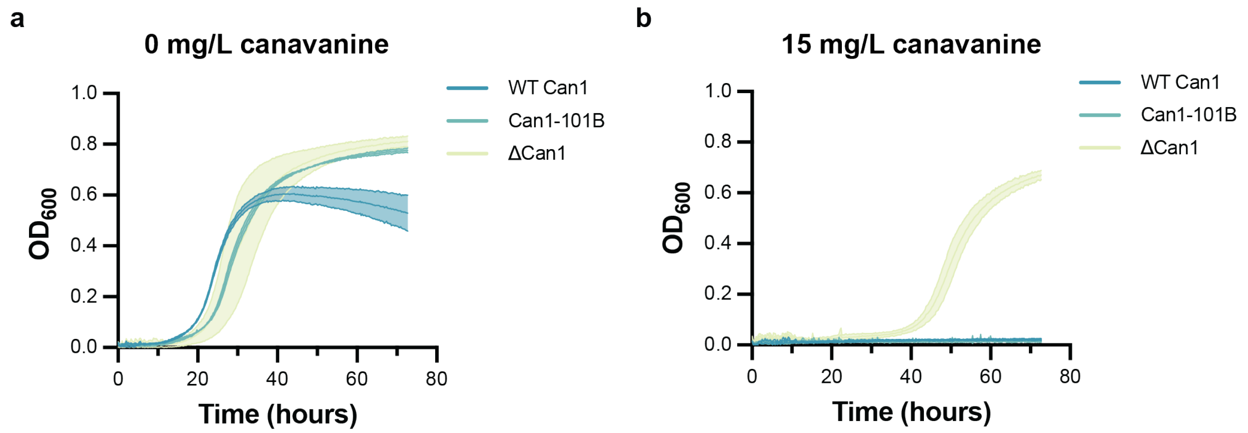

Figure S2. Can1 function is not impaired by the 101B tag used for LIVE-PAINT. (a) OD_{600} over 72 hours of three strains: wild-type (WT) in blue, LIVE-PAINT Can1 (Can1-101B) in teal, and Can1 deletion (ΔCan1) in lime green. The strains were grown in yeast synthetic media without arginine but with the sugars used for LIVE-PAINT imaging; 1% raffinose and 0.005% galactose. (b) OD_{600} over 72 hours of WT (blue), Can1-101B (teal), and ΔCan1 (lime green) strains grown in yeast synthetic media without arginine and with 1% raffinose and 0.005% galactose as used for LIVE-PAINT imaging. 15 mg/mL of the toxic arginine analog canavanine was also added to the media. Growth of all three strains without canavanine is similar; however only the ΔCan1 strain can grow in 15 mg/L canavanine. This indicates that Can1 C-terminally tagged to 101B functions similarly to WT as both can transport canavanine at a similar rate which prevents growth. Graphs in (a) and (b) show mean +/- SD. N = 3 for all strains and conditions apart from ΔCan1 in canavanine where N = 2.

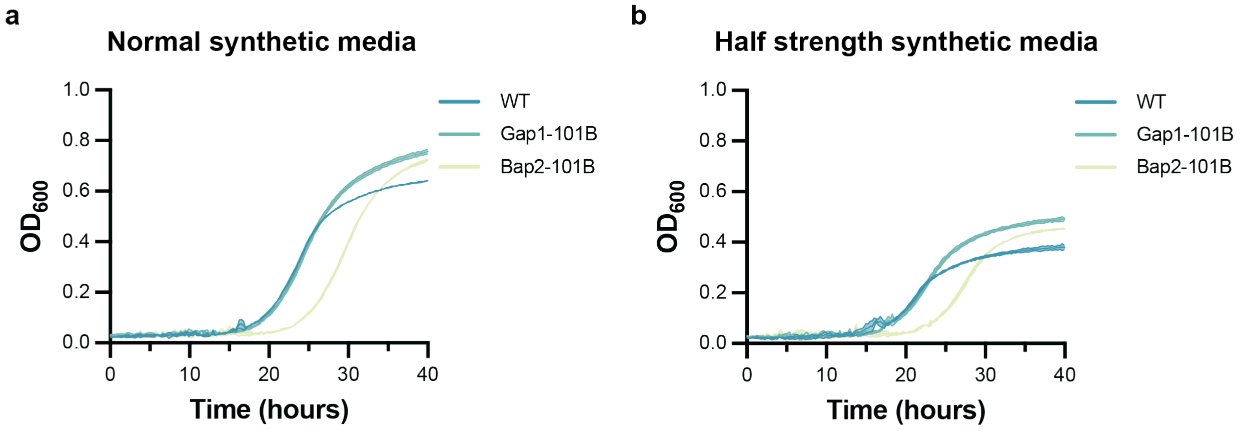

Figure S3. Gap1 and Bap2 function is not impaired by the 101B tag used for LIVE-PAINT. (a) OD_{600} over 40 hours of three strains: wild-type (WT) in blue, LIVE-PAINT Gap1 (Gap1-101B) in teal, and LIVE-PAINT Bap2 (Bap2-101B) in lime green. The strains were grown in yeast synthetic media with 1% raffinose and 0.005% galactose as used for LIVE-PAINT imaging. (b) OD_{600} over 40 hours of WT (blue), Gap1-101B (teal), and Bap2-101B (lime green) strains grown in half strength yeast synthetic media with 1% raffinose and 0.005% galactose. Growth of all three strains is similar in full strength and half strength synthetic media. This suggests that Gap1 and Bap1 C-terminally tagged to 101B function similar to WT as both can transport amino acids into the cell at a sufficient rate to support growth, even in low amino acid conditions. Bap2-101B is initially shows a slower growth rate in both conditions but reaches a similar OD_{600} to the other two strains by around 30 hours. Graphs in (a) and (b) show mean +/- SD. N = 3 for all strains.

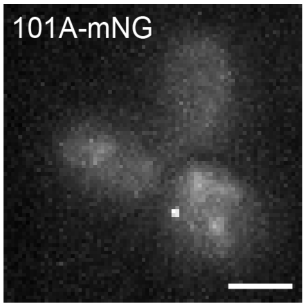

Figure S4. 101A-mNeonGreen alone does not localize to the plasma membrane. Diffraction-limited TIRF image of yeast expressing 101A-mNG but no protein tagged with 101B. No appreciable membrane localization is visible. Scale bar is 2 µm.

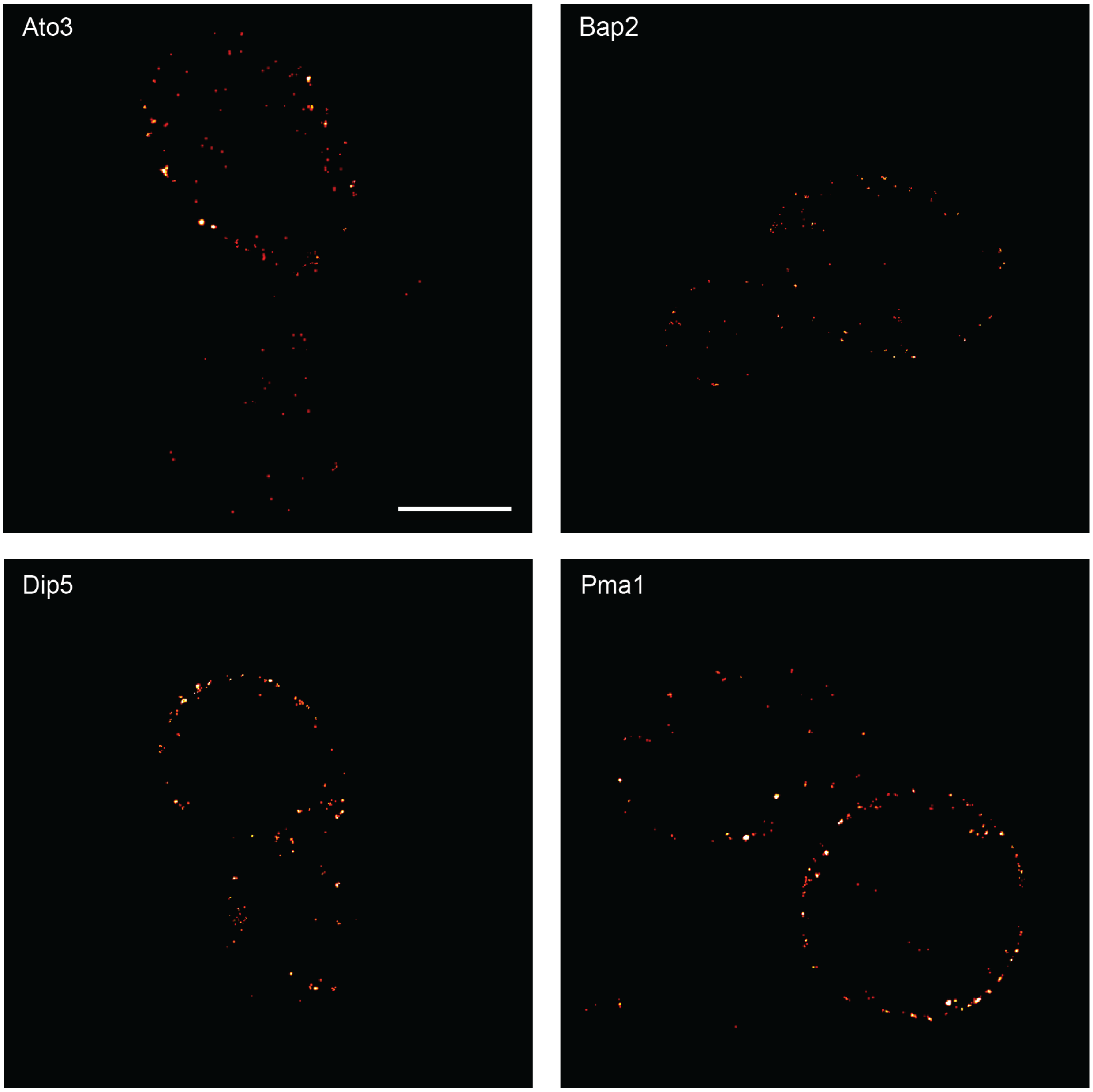

Figure S5. Full-cell super-resolution images of membrane proteins labelled with the 101A/B peptide pair shown in Figure 3. Scale bar is 2 μm and all images have the same dimensions.

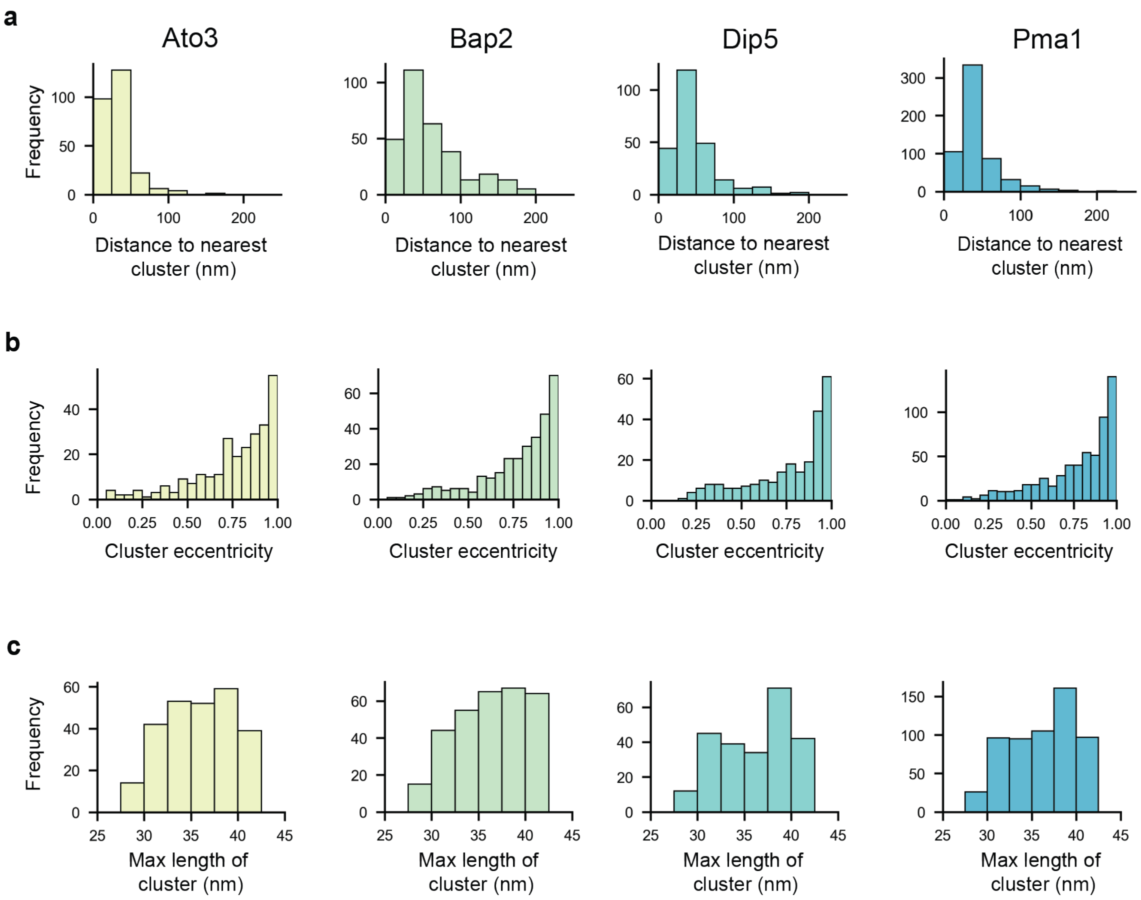

Figure S6. Analysis of whole population protein cluster data for each membrane protein imaged in Figure 3. Histograms showing the frequency distribution for all the protein clusters identified over all cells from SR images of Ato3, Bap2, Dip5, and Pma1 of: (a) the distance to the nearest cluster (nm); (b) the eccentricity of the clusters, (c) the maximum length of the clusters (nm).

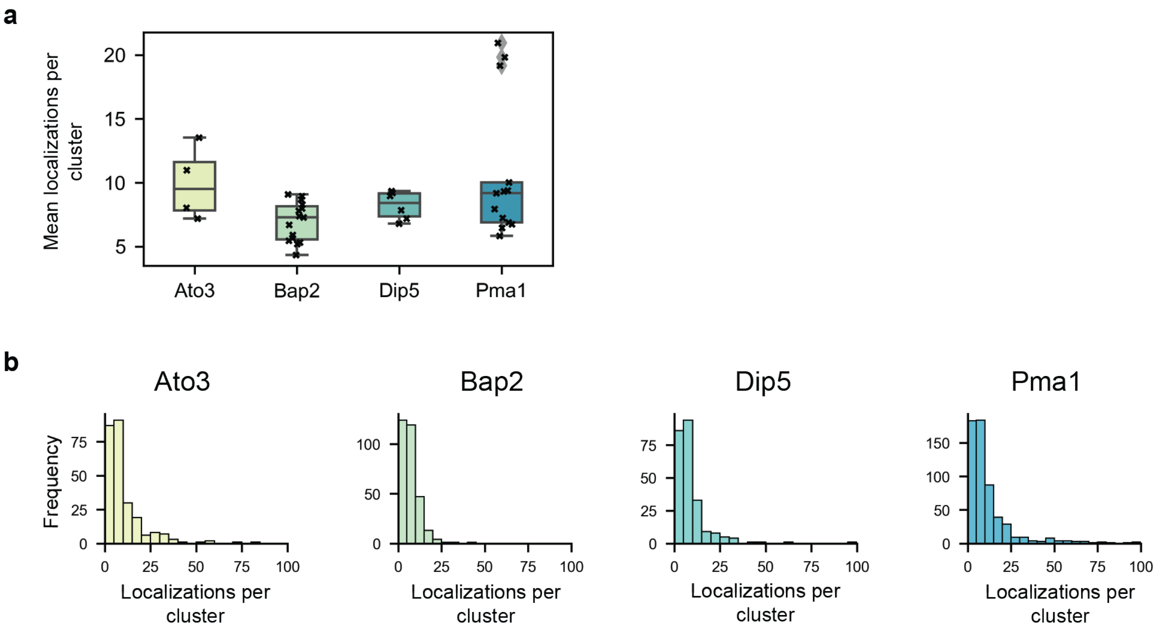

Figure S7. Quantification of the number of localizations per cluster for each membrane protein imaged in Figure 3. (a) Mean localizations per cluster for each cell. (b) Histograms showing the frequency distribution for all the protein clusters identified over all cells from SR images of Ato3, Bap2, Dip5, and Pma1 of the number of localizations in each cluster.

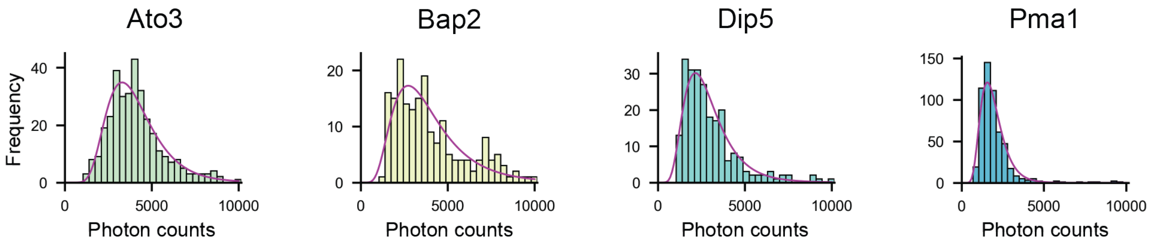

Figure S8. Histograms of photon counts for SR images of each membrane protein presented in Figure 3. All plots are log normal indicating that single molecules are localized during LIVE-PAINT super-resolution imaging. Log normal fits to the data are shown in magenta.

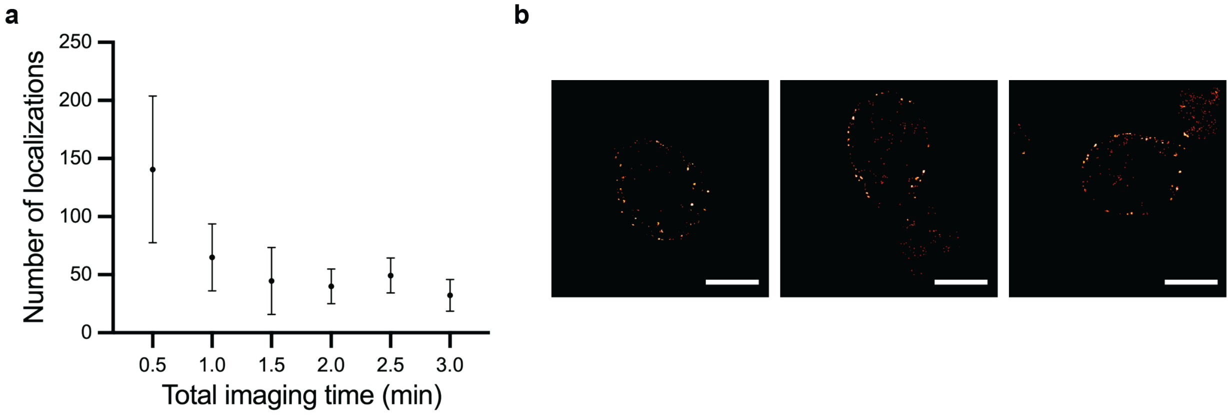

Figure S9. Localizations are detected throughout three minutes of imaging with LIVE-PAINT. (a) Plot showing the number of localizations identified with precision cut off 20 nm from SR images of Bap2 using the LIVE-PAINT technique binned over 30 second periods. Each point represents the mean over three cells and error bars represent SD. Localizations from neighboring cells in the same field of view were excluded. (b) Super-resolution images of the three cells whose localization data was used to produce the plot shown in (a). Scale bars are 2 μm.

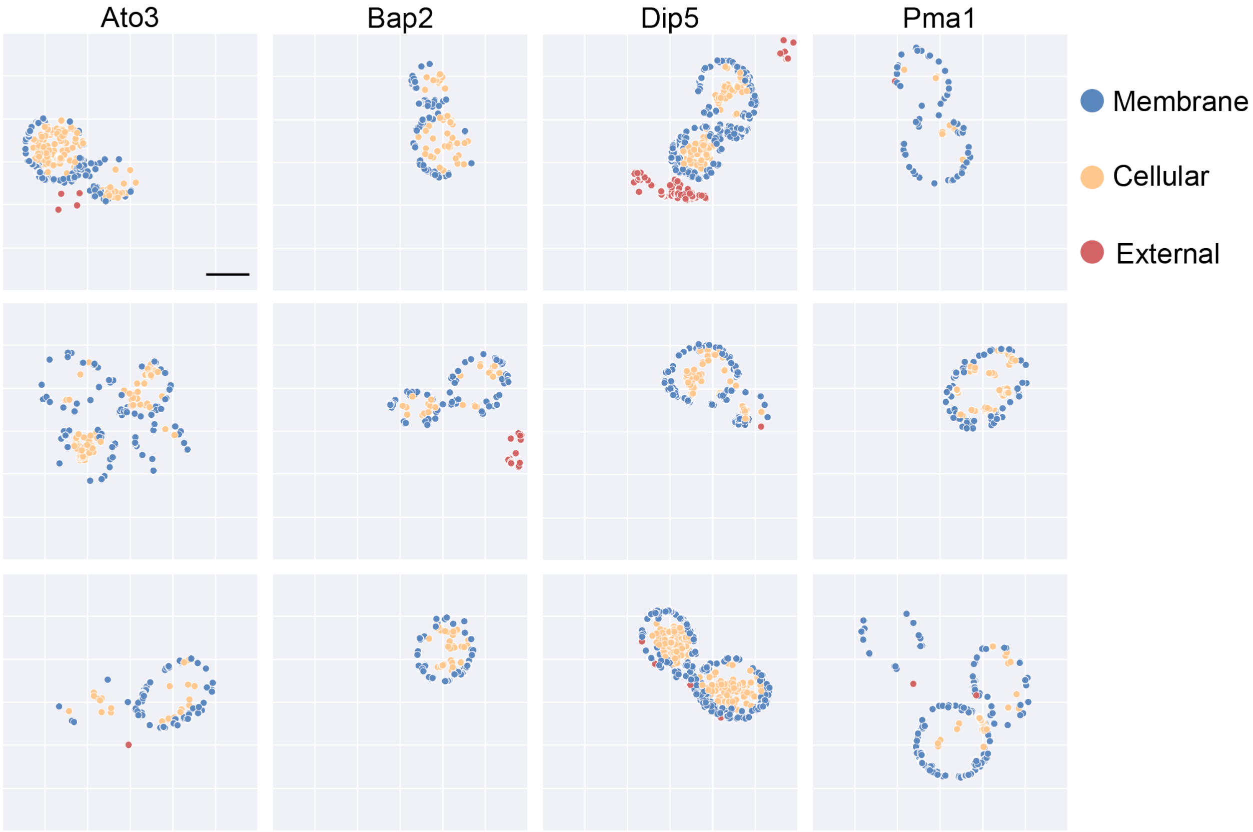

Figure S10. Representative plots showing localizations classified as membrane, cellular or external for membrane proteins labelled with the 101A/B peptide pair. Localizations classed as membrane are plotted in blue, cellular in yellow and external in red. Scale bar is 2 μm and all images have the same dimensions.

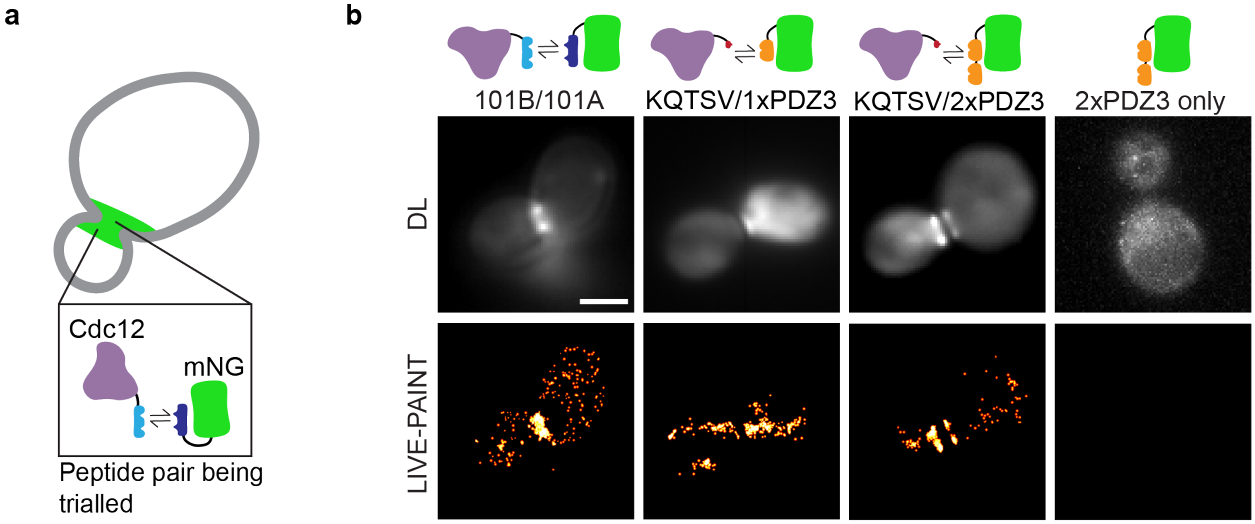

Figure S11. KQTSV can be used as a peptide label for LIVE-PAINT with two tandem repeats of PDZ3 fused to mNeonGreen. (a) Schematic representation of the localization of the septum protein Cdc12 in a dividing *S. Cerevisiae* cell and the LIVE-PAINT labelling strategy used to image this protein. (b) Diffraction-limited (DL) (top) and LIVE-PAINT (bottom) images of Cdc12 imaged by C-terminal labelling with 101B and co-expressing 101A-mNG or by C-terminal labelling with KQTSV and either co-expressing 1xPDZ3-mNG or 2xPDZ3-mNG and control images of 2xPDZ3-mNG only. Scale bar is 2 µm and all images have the same dimensions.

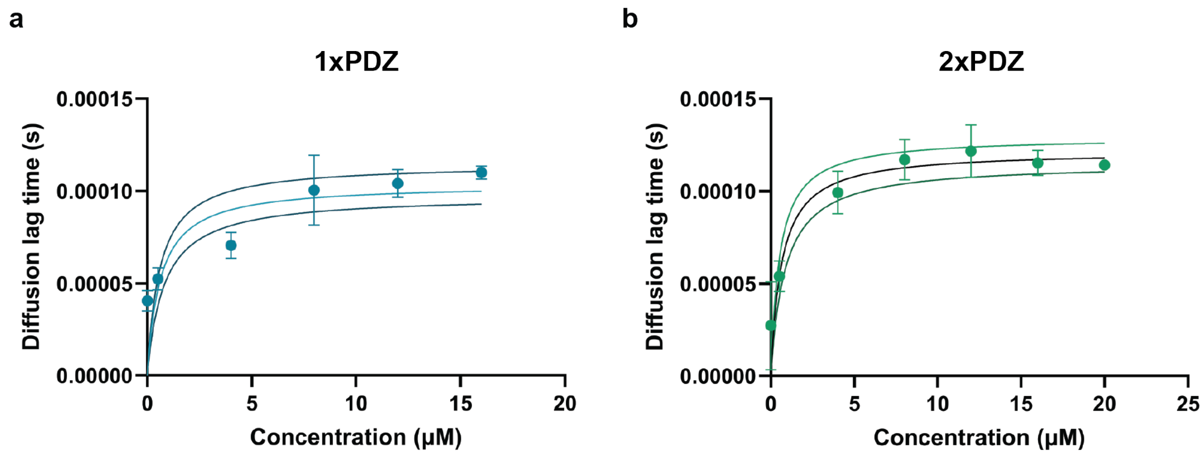

Figure S12. Graphs of diffusion lag time as a function of PDZ concentration for the 1xPDZ and 2xPDZ proteins binding to the KQTSV peptide. Diffusion lag time was measured at different concentrations of (a) 1xPDZ or (b) 2xPDZ when incubated with 100 nM of SeNBD labelled KQTSV peptide. By fitting the data using a non-linear regression function (binding kinetics, one site specific binding) the K_D was found to be 670 +/- 110 nM (mean +/- SD, n = 2) for 1xPDZ and 680 +/- 170 nM (mean +/- SD, n = 3) for 2xPDZ.

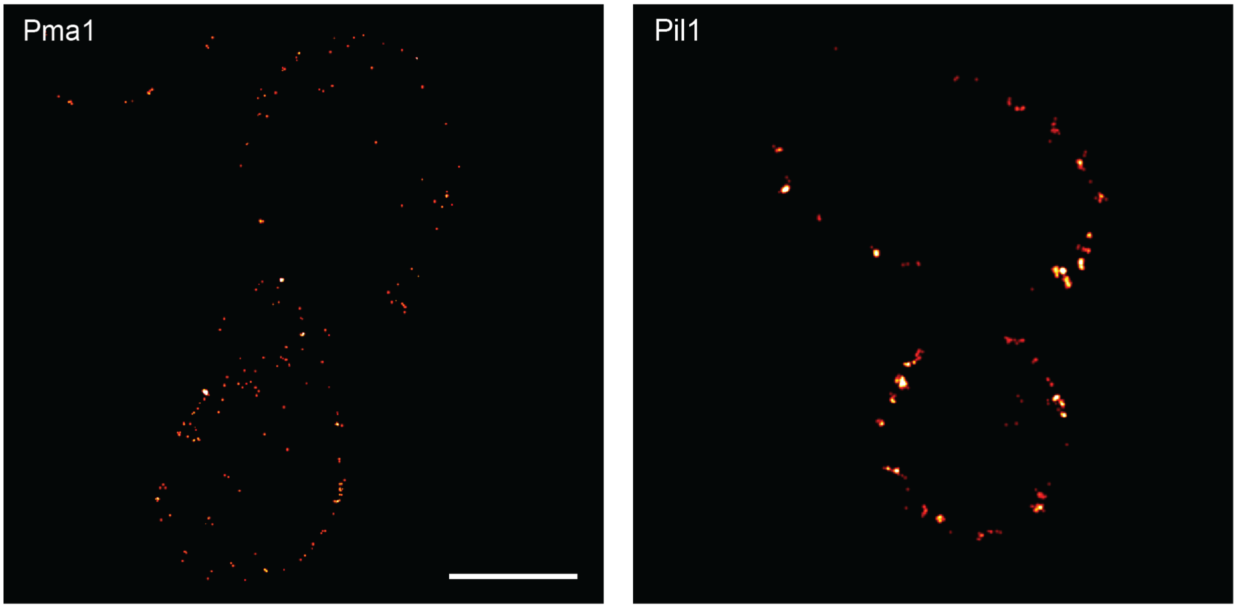

Figure S13. Full-cell super-resolution images of membrane proteins labelled with the KQTSV/2xPDZ3 peptide-protein pair shown in Figure 4. Scale bar is 2 μm and all images have the same dimensions.

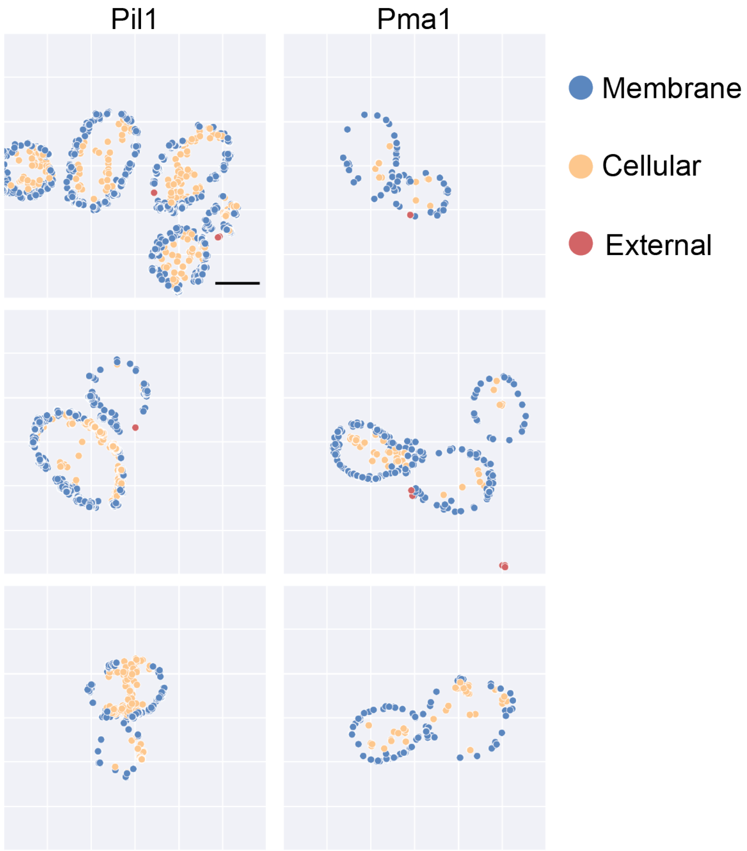

Figure S14. Representative plots showing localizations classified as membrane, cellular or external for membrane proteins labelled with the KQTSV/2xPDZ3 peptide-protein pair. Localizations classed as membrane are plotted in blue, cellular in yellow and external in red. Scale bar is 2 μm and all images have the same dimensions.

Table S1. Membrane transporter proteins tagged and imaged in this study.

Protein	Number of proteins/cell[9]	Protein function[9]
Ady2	1,298	Transporter protein required for ammonia export and acetate uptake and resistance.
Agp1	4,012	Broad substrate range permease that transports asparagine and glutamine with intermediate specificity.
Ato3	2,211	Transporter protein required for ammonia export.
Bap2	6,997	Permease for leucine, valine and isoleucine. Also transports cysteine, methionine, phenylalanine, tyrosine and tryptophan.
Can1	3,618	High-affinity permease for arginine.
Dip5	5,961	Can transport glutamate, aspartate, glutamine, asparagine, serine, alanine and glycine.
Fcy2	5,492	This permease has a broad specificity towards purines, and also transport cytosine and 5-methylcytosine but neither uracil nor thymine.
Gap1	3,634	General amino-acid permease involved in the uptake of all the naturally occurring L-amino-acids, related compounds such as ornithine and citrulline, some D-amino acids, toxic amino acid analogs such as azetidine-2-carboxylate, and the polyamines putrescine and spermidine.
Mup1	4,102	High affinity permease for methionine.
Pma1	40,732	The plasma membrane ATPase of plants and fungi is a hydrogen ion pump.
Ptr2	2,551	Uptake of small peptides.
Sit1	2,822	Involved in the transport of siderophore ferrioxamine B and so has a role in iron homeostasis.

Table S2. Summary statistics of super-resolution images of membrane associated proteins imaged using LIVE-PAINT in Figure 3.

**Protein**	**Number of Cells**	**Average Resolution (nm)**	**Average Precision (nm)**	**Average Number of Localizations**
Pma1	15	67.28 +/- 13.40	10.73 +/- 0.40	367 +/- 315
Bap2	15	61.40 +/- 15.03	10.56 +/- 1.00	207 +/- 95
Dip5	6	84.44 +/- 20.53	10.32 +/- 0.44	474 +/- 294
Ato3	6	73.46 +/- 24.55	10.69 +/- 1.18	492 +/- 565

Resolution was calculated using FRC and all values are shown as mean +/- SD.

Table S3. Summary statistics of super-resolution images of membrane associated proteins imaged using LIVE-PAINT and the peptide-protein pair KQTSV/2xPDZ3 in Figure 4.

**Protein**	**Number of Cells**	**Average Resolution (nm)**	**Average Precision (nm)**	**Average Number of Localizations**
Pma1	5	113.61 +/- 52.70	10.93 +/- 0.21	204 +/- 104
Pil1	5	101.19 +/- 17.12	20.5 +/- 5.88	1157 +/- 718

Resolution was calculated using FRC and all values are shown as mean +/- SD.

Table S4. Summary statistics of super-resolution images of Arc35 and Pil1 imaged using two color LIVE-PAINT and shown in Figure 5.

**Protein**	**Fluorescent protein**	**Average Resolution (nm)**	**Average Precision (nm)**	**Average Number of Localizations**
Arc35	mNG	83.03 +/- 21.51	15.59 +/- 3.61	657 +/- 475
Pil1	mCherry	72.45 +/- 27.94	19.73 +/- 2.87	620 +/- 380

Resolution was calculated using FRC and all values are shown as mean +/- SD. Number of cells = 6.

Table S5. Constructs used to measure binding kinetics *ex vivo*.

**Name (Sequence)**	**Mass ($gmol^{-1}$)**
PDZ peptide (SeNBDlinker-KQTSV)	901
1xPDZ<br>(MGLNDIFEAQKIEWHEGSGEEDIPREPRRIVIHRGSTGLGFNIVGGEDGEGIFISFILAGGPADLSGELRKGDQILSVNGDLRNASHEQAAIALKNAGQTVTIIAQYKPEEYSRFEAGSHHHHHH*)	13,800
2xPDZ<br>(MGLNDIFEAQKIEWHEGSGEEDIPREPRRIVIHRGSTGLGFNIVGGEDGEGIFISFILAGGPADLSGELRKGDQILSVNGDLRNASHEQAAIALKNAGQTVTIIAQYKPEEYSRFEAGGSGSGLQGEEDIPREPRRIVIHRGSTGLGFNIVGGEDGEGIFISFILAGGPADLSGELRKGDQILSVNGDLRNASHEQAAIALKNAGQTVTIIAQYKPEEYSRFEAGSHHHHHH*)	25,100

Table S6. Primers for tagging membrane proteins at their genomic loci.

**Name**	**Sequence**
p6h_int_F	CTAATCCAAGGAGGTTTACGGACCAGGGGAACTTTCCAGATTCAGAAGCTTCGTACGCTGCA
p6h_int_R	CATGAAAAATTAAGAGAGATGATGGAGCGTCTCACTTCAAACGCAGGCGTTAGTATCGAATCG
ADY2_F	TCGTCCATTCCCATTACCATCTACTGAAAGGGTAATCTTTGGTGGATCAGGCTCTGG
ADY2_R	TTTTTATTTCAATAGTTCTCGTTATTAGTAGGTCGTGCTCATTAGAAAAACTCATCGAGCATC
AGP1_F	GAACGGACCTTATTGGAAAAGGGTCGTTGCCTTCTGGTGTGGTGGATCAGGCTCTGG
AGP1_R	CAAAAATGAATAAATATAAAAGAAGTAAATGCTTTTTTTTATTAGAAAAACTCATCGAGCATC
ATO3_F	TTATTTAGCCTTCAGGGCGCACACAATGCCAAATGCTCCTGGTGGATCAGGCTCTGG

ATO3_R	TTTTAATGTTTTATAAGTTTTGTTTTTCATTTCATACCCTATTAGAAAAACTCATCGAGCATC
BAP2_F	GAATATGTCTTTGATGAGAAAAGCTTATCATTTCTGGTGTGGTGGATCAGGCTCTGG
BAP2_R	TCTAATGGGAAGTGTCCAGACCTGAGTGGTGTAGTTAAGTATTAGAAAAACTCATCGAGCATC
CAN1_F	ACCAAAGACTTTTTGGGACAAATTTTGGAATGTTGTAGCAGGTGGATCAGGCTCTG
CAN1_R	ATGGCGTGGAAATGTGATCAAAGGTAATAAAACGTCATATATGGCGGCGTTAGTATC
DIP5_F	TATGGAGTGGTTCTATGAAAAATTTTTGGGTAATATCTTCGGTGGATCAGGCTCTG
DIP5_R	ATAGTTTCATGTTGCCCTAAGGCTTTGATTAAAAAGGCATATGGCGGCGTTAGTATC
FCY2_F	CAACATCTTAAGACCTTTAGAATTAAAATACTTCGGTCGTGGTGGATCAGGCTCTG
FCY2_R	GAAATGTGCACGGGGAAATGATCGCCCTAATCATTACTTCATGGCGGCGTTAGTATC
GAP1_F	CACAAAGCCAAGATGGTATAGAATCTGGAATTTCTGGTGTGGTGGATCAGGCTCTG
GAP1_R	ATCTAAAAAATAAAGTCTTTTTTTGTCGTTGTTCGATTCAATGGCGGCGTTAGTATC
MUP1_F	TATAATCGAACATTACAAAAGTGAACAAGAAAAATCGCTGGGTGGATCAGGCTCTG
MUP1_R	GATTATAAGAATCGAGATGAGATGGTAAGTACCTTTTTGGATGGCGGCGTTAGTATC
PMA1_F	TGCTATGCAAAGAGTCTCTACTCAACACGAAAAGGAAACCGGTGGATCAGGCTCTG
PMA1_R	AATGTGACAAAAATTATGATTAAATGCTACTTCAACAGGAATGGCGGCGTTAGTATC
PMA1_URA3_R	AATGTGACAAAAATTATGATTAAATGCTACTTCAACAGGATCAGTTTTGCTGGCCGC

PTR2_F	ATTAGAACCAATGGAAAGTCTAAGATCCACCACCAAATATGGTGGATCAGGCTCTG
PTR2_R	AAAAAAAAAAAAAAAAAAGACAGTAAGTTAATTAAACGCAATGGCGGCGTTAGTATC
SIT1_F	ATTCTTTACGCACTTTACAAGCAGTAAAGATAGGAAAGATGGTGGATCAGGCTCTG
SIT1_R	GCTATATGTGCATGTATGAAATTATTTGGGTGAGATAATAATGGCGGCGTTAGTATC

p6h_int_F and p6h_int_R were used to amplify the plasmid containing pGAL1 101A-mNG, pGAL1 PDZ3-mNG, or pGAL1 2xPDZ3-mNG and HIS3 selection marker and to integrate it into the genome replacing GAL2. PMA1_URA3_R was used with PMA1_F to amplify the plasmid containing the KQTSV peptide and the URA3 selection marker. The ADY2, AGP1, ATO3, and BAP3 primers were used to amplify the plasmid containing the 101B peptide and the G418 selection marker. All other primers were used to amplify the plasmid containing the 101B peptide and the URA3 selection marker. The primer name contains the name of the gene being tagged and the primers come in pairs, with the "_F" and "_R" primers being used as forward and reverse primers.

Table S7. Primers for generating a yeast strain with two membrane-associated proteins tagged at their genomic loci.

Name	Sequence
ARC35_F	ACAGGCAAGAAGAACCTTCACCGGTAGAAAGATTGTCTACGGTGGATCAGGCTCTGG
ARC35_R	TAACCCTTTTTACGGATTCTTACGTACTTATTTAATCTTTATTAGAAAAACTCATCGAGCATC
PIL1_MULTI_F	ACACCAGCAAAGTGAGTCTCTTCCCCAACAAACAACAGCTGGTGGATCAGGCTCTGG
PIL1_MULTI_R	TTTTTTTTTTTGTTTCTAATAGATTGTTGATTTATTTTGAGGCGTTAGTATCGAATCGAC

The primers come in pairs, with the "_F" and "_R" primers being used as forward and reverse primers. Primers ARC35_F/R were used to fuse 101B to Arc35 by amplifying the plasmid containing 101B and the G418 selection marker. PIL1_MULTI_F/R were used to fuse 108B to PIL1 and insert pGAL1 101A-mCherry at the same gentic loci by amplifying the plasmid containing 108B, pGAL1-108A-mCherry and the selection marker LEU2.

Table S8. List of yeast strains used in this study.

#	Gene	Coiled-coil pair	Genotype
1	ADY2	101A/B	MATa his3Δ1 leu2Δ0 met15Δ0 ura3Δ0 gal2Δ::HIS3MX6 pGAL1 101A-mNeonGreen ADY2-101B::KANMX6
2	AGP1	101A/B	MATa his3Δ1 leu2Δ0 met15Δ0 ura3Δ0 gal2Δ::HIS3MX6 pGAL1 101A-mNeonGreen AGP1-101B::KANMX6
3	ATO3	101A/B	MATa his3Δ1 leu2Δ0 met15Δ0 ura3Δ0 gal2Δ::HIS3MX6 pGAL1 101A-mNeonGreen ATO3-101B::KANMX6
4	BAP2	101A/B	MATa his3Δ1 leu2Δ0 met15Δ0 ura3Δ0 gal2Δ::HIS3MX6 pGAL1 101A-mNeonGreen BAP2-101B::KANMX6
5	CAN1	101A/B	MATa his3Δ1 leu2Δ0 met15Δ0 ura3Δ0 gal2Δ::HIS3MX6 pGAL1 101A-mNeonGreen CAN1-101B::URA3
6	DIP5	101A/B	MATa his3Δ1 leu2Δ0 met15Δ0 ura3Δ0 gal2Δ::HIS3MX6 pGAL1 101A-mNeonGreen DIP5-101B::URA3
7	FCY2	101A/B	MATa his3Δ1 leu2Δ0 met15Δ0 ura3Δ0 gal2Δ::HIS3MX6 pGAL1 101A-mNeonGreen FCY2-101B::URA3
8	GAP1	101A/B	MATa his3Δ1 leu2Δ0 met15Δ0 ura3Δ0 gal2Δ::HIS3MX6 pGAL1 101A-mNeonGreen GAP1-101B::URA3
9	MUP1	101A/B	MATa his3Δ1 leu2Δ0 met15Δ0 ura3Δ0 gal2Δ::HIS3MX6 pGAL1 101A-mNeonGreen MUP1-101B::URA3
10	PMA1	101A/B	MATa his3Δ1 leu2Δ0 met15Δ0 ura3Δ0 gal2Δ::HIS3MX6 pGAL1 101A-mNeonGreen PMA1-101B::URA3
11	PTR2	101A/B	MATa his3Δ1 leu2Δ0 met15Δ0 ura3Δ0 gal2Δ::HIS3MX6 pGAL1 101A-mNeonGreen PTR2-101B::URA3
12	SIT1	101A/B	MATa his3Δ1 leu2Δ0 met15Δ0 ura3Δ0 gal2Δ::HIS3MX6 pGAL1 101A-mNeonGreen SIT1-101B::URA3
13	PMA1	2xPDZ3/ KQTSV	MATa his3Δ1 leu2Δ0 met15Δ0 ura3Δ0 gal2Δ::HIS3MX6 pGAL1 PDZ3-mNeonGreen PMA1-KQTSV::URA3

14	PIL1	2xPDZ3/ KQTSV	MATa his3Δ1 leu2Δ0 met15Δ0 ura3Δ0 gal2Δ::HIS3MX6 pGAL1 PDZ3-mNeonGreen PIL1-KQTSV::URA3
15	ARC35 & PIL1	101A/B and 108A/B	MATa his3Δ1 leu2Δ0 met15Δ0 ura3Δ0 gal2Δ::HIS3MX6 pGAL1 101A-mNeonGreen ARC35-101B::KANMX6 Pil1-108B pGAL1 108A-mCherry::LEU2

The gene being tagged is listed in the "Gene" column and the full genotype of the strain used for imaging is given in the "Genotype" column.

References

(1) Guthrie, C.; Fink, G. R. *Guide to Yeast Genetics and Molecular and Cell Biology, Part C*; Gulf Professional Publishing, 2002.

(2) Thompson, K. E.; Bashor, C. J.; Lim, W. A.; Keating, A. E. SYNZIP Protein Interaction Toolbox: In Vitro and in Vivo Specifications of Heterospecific Coiled-Coil Interaction Domains. *ACS Synth. Biol.* **2012**, *1* (4), 118–129. https://doi.org/10.1021/sb200015u.

(3) Oi, C.; Gidden, Z.; Holyoake, L.; Kantelberg, O.; Mochrie, S.; Horrocks, M. H.; Regan, L. LIVE-PAINT Allows Super-Resolution Microscopy inside Living Cells Using Reversible Peptide-Protein Interactions. *Commun. Biol.* **2020**, *3* (1), 1–10. https://doi.org/10.1038/s42003-020-01188-6.

(4) Hawkins, K. M.; Smolke, C. D. The Regulatory Roles of the Galactose Permease and Kinase in the Induction Response of the GAL Network in Saccharomyces Cerevisiae. *J. Biol. Chem.* **2006**, *281* (19), 13485–13492. https://doi.org/10.1074/jbc.M512317200.

(5) Stirling, D. R.; Swain-Bowden, M. J.; Lucas, A. M.; Carpenter, A. E.; Cimini, B. A.; Goodman, A. CellProfiler 4: Improvements in Speed, Utility and Usability. *BMC Bioinformatics* **2021**, *22* (1), 433. https://doi.org/10.1186/s12859-021-04344-9.

(6) Sander, J.; Ester, M.; Kriegel, H.-P.; Xu, X. Density-Based Clustering in Spatial Databases: The Algorithm GDBSCAN and Its Applications. *Data Min. Knowl. Discov.* **1998**, *2* (2), 169–194. https://doi.org/10.1023/A:1009745219419.

(7) de Moliner, F.; Konieczna, Z.; Mendive-Tapia, L.; Saleeb, R. S.; Morris, K.; Gonzalez-Vera, J. A.; Kaizuka, T.; Grant, S. G. N.; Horrocks, M. H.; Vendrell, M. Small Fluorogenic Amino Acids for Peptide-Guided Background-Free Imaging. *Angew. Chem. Int. Ed.* **2023**, *62* (4), e202216231. https://doi.org/10.1002/anie.202216231.

(8) Chappard, A.; Leighton, C.; Saleeb, R. S.; Jeacock, K.; Ball, S. R.; Morris, K.; Kantelberg, O.; Lee, J.-E.; Zacco, E.; Pastore, A.; Sunde, M.; Clarke, D. J.; Downey, P.; Kunath, T.; Horrocks, M. H. Single-Molecule Two-Color Coincidence Detection of Unlabeled Alpha-Synuclein Aggregates. *Angew. Chem. Int. Ed.* **2023**, *62* (15), e202216771. https://doi.org/10.1002/anie.202216771.

(9) Cherry, J. M.; Hong, E. L.; Amundsen, C.; Balakrishnan, R.; Binkley, G.; Chan, E. T.; Christie, K. R.; Costanzo, M. C.; Dwight, S. S.; Engel, S. R.; Fisk, D. G.; Hirschman, J. E.; Hitz, B. C.; Karra, K.; Krieger, C. J.; Miyasato, S. R.; Nash, R. S.; Park, J.; Skrzypek, M. S.; Simison, M.; Weng, S.; Wong, E. D. Saccharomyces Genome Database: The Genomics Resource of Budding Yeast. *Nucleic Acids Res.* **2012**, *40* (Database issue), D700. https://doi.org/10.1093/nar/gkr1029.
